# A New Conceptual ‘Cylinder’ Framework for Sustainable Bioeconomy Systems and Their Actors

**DOI:** 10.1007/s10806-021-09850-7

**Published:** 2021-04-01

**Authors:** Hugo de Vries, Mechthild Donner, Monique Axelos

**Affiliations:** 1 INRAE—French National Research Institute for Agriculture, Food and Environment, UMR IATE (University Montpellier, INRAE, Institut Agro), 2 Place Pierre Viala, 34060 Montpellier, France; 2 INRAE—French National Research Institute for Agriculture, Food and Environment, UMR MOISA (University Montpellier, INRAE, Cirad, Ciheam-Iamm, Institut Agro, IRD), 2 Place Pierre Viala, 34060 Montpellier, France; 3 INRAE—French National Research Institute for Agriculture, Food and Environment, 147 Rue de l’Université, Paris, France

**Keywords:** Sustainable bioeconomy, Conceptual framework, Complex adaptive systems, Game theory, Business cases

## Abstract

Concepts for sustainable bioeconomy systems are gradually replacing the ones on linear product chains. The reason is that continuously expanding linear chain activities are considered to contribute to climate change, reduced biodiversity, over-exploitation of resources, food insecurity, and the double burden of disease. Are sustainable bioeconomy systems a guarantee for a healthy planet? If yes, why, when, and how? In literature, different sustainability indicators have been presented to shed light on this complicated question. Due to high degrees of complexity and interactions of actors in bioeconomy systems, trade-offs and non-linear outcomes became apparent. This fueled the debates about the normative dimensions of the bioeconomy. In particular, the behavior of actors and the utilization of products do not seem to be harmonized according to the environmental, social, and economic pillars of sustainability. Potential conflicts require a new conceptual framework that is here introduced. It consists of a ‘sustainability’ cylinder captured between an inner-cylinder, representing order, and an outer-cylinder for chaos, based on the laws of physics and complex adaptive systems. Such a framework permits (bioeconomy) systems to propagate in the sustainability zone only if they follow helical pathways serving as the new norms. Helices are a combination of two sinusoidal patterns. The first represents here the sustainable behavior of interacting actors and the second the balanced usage of resources and products. The latter counteracts current growth discourses. The applicability of the conceptual cylinder framework is positively verified via 9 cases in Europe, which encompass social-organizational and product-technological innovations. –

## Introduction

In 2018, the European circular and sustainable bioeconomy strategy (Patermann & Aguilar, 2018; EC DG R&I, 2018) and the circular bio-society 2050 reports (Biosociety, 2020) provide clear, largely supported, ambitious visions for bioeconomy systems. They are supposed to contribute to the highly challenging Sustainable Development Goals (SDG) of the United Nations (Lu et al., 2015). Recently, the European New Green Deal and Fork-to-Farm strategy have been setting the scene for the new research and innovation programs for the bioeconomy, including the food domain (EC, 2020a; EC, 2020b; EC, 2020c).

These strategies are responses to the current linear ‘take – make – consume – dispose of’ chains, which are associated with mass consumption and unsustainable behavior of actors. These chains often result in waste (Fusions, 2019; Morone, 2019), over-exploitation of natural resources (Meadows et al., 1972; EC, 2011,1), and competing claims for resources (Tuck et al., 2012), food and nutritional insecurity. Numerous indicators show (exponential) growth patterns (e.g. for climate change, biodiversity loss, overweight and obesity, energy, and water usage, urbanization, social inequalities), possibly with severe consequences for the planet, profits, and the well-being of people (Barnosky et al., 2012; IPCC, 2019; IPBES, 2019; SAPEA, 2020). The importance of these challenges is reflected by their prominence in most of the 17 SDGs and even more accentuated by the current COVID-19 crisis.

Systems showing continuous overall (exponential) growth patterns tend to end up in chaos or order as explained by the laws of thermodynamics (Georgescu-Roegen, 1971; Prigogine & Stengers, 1985) via a series of small and large scale events following power laws, like avalanches on a growing sand pile (Andriani & McKelvey, 2011; Bak et al., 1988). The zone in between order and chaos, called the melting zone (Carbonara et al., 2010), is narrow, however, with a certain bandwidth. One can hypothesize that systems continuously evolving in this zone are sustainable by nature, as further discussed below. This should also hold for bioeconomy systems that are here defined as systems that organize the utilization of bio-based products (including natural resources) within certain boundaries, appreciating its wider environmental, social, political, and economic context.

Sustainability and sustainable bioeconomy systems (SBS) are now widely discussed (e.g. Dubois and Gomez, 2016; Bugge et al., 2016; D'Amato et al., 2017; Szekacs, 2017), utilizing the Brundtland definition as the basis (WCED, 1987); hence, they integrally consider the three pillars ‘planet, people and profit’ and specific actions that will not compromise future generations. However, the understanding of the functioning of bioeconomy systems and the way to change the current trends are less obvious, in particular at the Global and European levels but also local levels. The appealing concept of a circular economy, as re-introduced by the Ellen MacArthur Foundation (2015), has widely been promoted in the past decade. It largely emerged from legislation (Murray et al., 2015), even though closed-loop thinking already exists since the beginning of agricultural production by feeding back essential nutrients to the soil. The success of the circular bioeconomy approaches will depend on transformation costs (like money, water, energy, and safety measures) for the recuperation and utilization of nutrients somewhere in the bioeconomy. To establish whether or not environmental sustainability is also at stake, a sound scientific basis is required e.g. based on thermodynamics and system boundaries (Korhonen et al., 2018). Social sustainability deserves attention as well (Ostrom, 2009). Here, we like to remark that the mathematical representation of a circle implies (i) ‘equally *fair and just* distances’ for all actors at the circle to its center and (ii) the absence of a beginning or end, hence *shared responsibilities* for all being suppliers and demanders at the circle. In contrast, traditional linear food and other fossil-based product chains, from producers, manufacturers, retailers to consumers, generally show end-of-chain dominance.

SBS needs metrics and joint actions to understand and measure the impacts of current actions (Bracco et al., 2019; Ronzon & M’Barek, 2018). Besides, they require resources, multi-level and landscape-oriented approaches (Geels, 2002); the landscape refers to the overall socio-technical setting for interactions of actors. Finally, they ask for renewed considerations of planetary boundaries presented as radars (Rockström et al., 2009); the boundaries are associated with the planet’s biophysical subsystems or processes. An alternative image is a Doughnut in which also social limits are incorporated (Raworth, 2017); the image of a doughnut shows an inner circle representing the ‘social foundation’ and the outer circle serving as the ‘ecological ceiling (or planet’s bio-physical limits)’. Beyond the ecological ceiling, one can speak about unacceptable environmental degradation, while below the social foundation the human primary needs are fundamentally challenged.

**Fig. 1:**
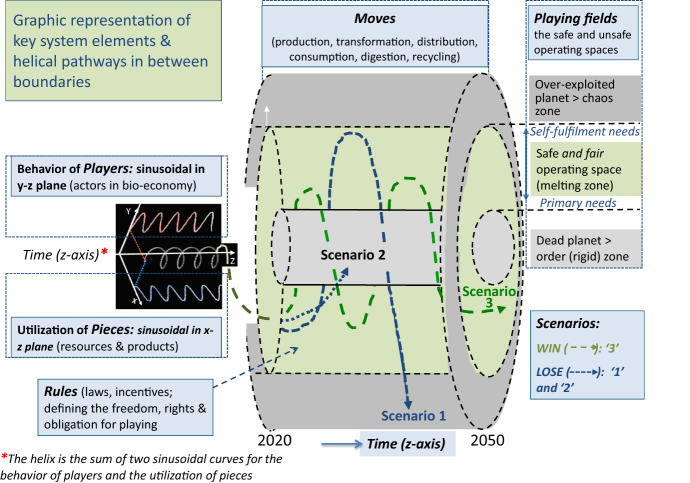
The cylinder confinement for sustainable bioeconomy (incl. food) systems evolving in the safe and fair operating space (SFOS), in between order and chaos, hence following scenario 3; the SFOS is identical to the melting zone in Fig. 3 in which Complex Adaptive Systems evolve. Bioeconomy (including food) systems that are unsustainable enter either in the chaos or order zone, following respectively the scenarios 1 or 2. Note the interconnectedness, via a helix, between the behavior of players and biomass utilization in a sustainable way. *Source:* Modified image of https://www.radartutorial.eu/06.antennas/pic/zirkulanim.gif is included

Radars and doughnuts partially provide information about the spaces within which human beings can act. In the case of planetary, environmental, boundaries only upper limits are given, while for social boundaries only lower limits are presented. Here, we state that one should also define lower limits for planetary boundaries and upper limits for social ones; however also for economic ones. This results in sets of social – or socio-economic – foundations and ceilings as well as ecological foundations and ceilings. As we will see later, this corresponds with the thoughts about correct interpretations of the order-melting-chaos zones. The chaos zone corresponds then to the zone beyond these ceilings, while the order zone is below the foundations. It should also be noted that radar and doughnut are presented as two-dimensional images, hence they are explicitly lacking the time dimension. This does not make them suitable for visualizing dynamic behavior and (un)sustainable outcomes in time. These two considerations will form the basis for constructing our conceptual ‘cylinder’ framework (see Fig. 1).

We hypothesize and discuss that sustainable (bioeconomy) systems are characterized by their balancing evolution between order and chaos, hence between social-environmental-economic foundations and ceilings. However, this immediately asks for a reflection on the normative dimension of sustainability, which is inherent in its conceptions and debates but very complex, as it involves different (conflicting) visions among various societal actors and contested pathways (Schlaile et al., 2017). The normative dimension also refers to the question of ethical leadership (Blok, 2018) in bioeconomy systems as well as of dedicated innovation systems (Pyka, 2017) and responsible research and innovation pathways (de Saille, 2015; von Schomberg, 2013). Without such a reflection, bioeconomy systems may pass ceilings and foundations. Also, the visions on desirable ceilings and foundations as well as on the pathways to sustainability may be quite different (Schlaile et al., 2017). This immediately poses the following questions: (a) In what way can the three dimensions of sustainability (environmental, social, and economic) be harmonized to avoid conflicting visions and outcomes of bioeconomy systems? (b) Which interacting system elements propagate in a sinusoidal manner under changing conditions? (c) Which system elements provide the ‘environments’ and ‘limits’ such that balancing behavior may appear? (e) Which system elements reveal (un) sustainable pathways? (f) Which real-life cases provide insights and allow formulating policy options?

To answer these questions, we will stepwise introduce a conceptual framework for understanding and analyzing SBS in the following section. First, a cylinder concept is constructed for dynamic systems that encompass all system elements (playing fields, players, pieces, moves, rules, win/lose, and duration). It includes the notion that sustainable systems continuously evolve in a melting zone in between order and chaos, hence following combined helical pathways. Second, it incorporates the mathematical description of helices as constructs of 2 sinusoidal-like waves. In bioeconomy, this brings the behavior of players and the utilization of products in harmony. Also, both the overall global bioeconomy system and the multiple local systems should be able to perform sustainably. Third, the theory of complex adaptive systems – i.e. systems that continuously evolve in the ‘melting’ zone also if challenged by extreme conditions – is linked with the conceptual framework, thus revealing which player networks proceed harmonically in the melting zone. The same should hold for the utilization of products. Here, we take into account that the earth is an open thermodynamic system, i.e. fueled by solar energy input and emission outputs of greenhouse gases. In the ‘case studies’ section, the conceptual framework is elucidated via practical cases. In the ‘discussion’ section, the framework serves the debates on the normative dimensions of SBS and its actors. Finally, conclusions are drawn and options are given how stakeholders, including policymakers, could act while operating within the here proposed normative dimensions of a sustainable bioeconomy.

## Towards a Conceptual Framework for Analyzing Sustainable Bioeconomy Systems (SBS)

### From Radar and Doughnut to a Cylinder Confinement

Which ‘confinement’ area allows systems to follow sinusoidal-like patterns? We start with the integration of the time dimension in the previously presented ‘doughnut’ and ‘radar’. This yields a novel cylinder configuration with three zones (Fig. 1), the order, melting, and chaos zones. The melting zone is a hollow cylindrical tube centered around a ‘highly ordered (rigid) zone’. This inner cylinder represents e.g. the non-vital planet earth that is not able to respond to primary needs. A third, outer, cylinder represents a ‘highly chaotic zone’, e.g. the over-exploited or over-heated planet earth where excessive behavior leads to extreme and irreversible inequalities. Consequently, the melting zone can also be called a safe operating space in social-ecological systems (Anderies et al., 2019; Rockström et al., 2009) or *fair and safe operating space* (this publication); the latter refers to the reflections on framings of food as a commodity, commons, and human right (SAPEA, 2020). The cylinder configuration allows defining contrasting *outcomes* – presented as 3 different scenarios – for bioeconomy systems (‘win-lose’ in game theory; like SDG outcomes). Besides, the *other 6 key elements of systems or game theory* (Neumann & Morgenstern, 1944) are presented, namely players, pieces, rules, moves, playing fields, and duration/time (the light blue text boxes).

The element ‘players’ include all actors in bioeconomy systems (like producers, NGOs, public institutions, citizens); their overall behavior should follow pathways in the green area to reach a sustainable output. The element ‘pieces’ includes renewable resources, transformed functional and healthy products, recycled products, and services like safety & security & health insurance, labor, etc. Overall, their utilization should also follow pathways in the green area. The element ‘moves’ refers to all steps dealing with the handling of pieces including production, manufacturing, distribution, consumption, usage/digestion, and recycling; the outcomes of moves should guarantee that pathways remain in the green area. The element ‘playing fields’ regards all kinds of territories from planetary scales to local environments (geographic, political, legislative, etc.). These should be chosen such that pathways can be followed in the green area. The element ‘rules’ encompasses legislation and incentives, hence defines the freedom to play, the rights, and the obligations for players to reach sustainable outcomes. The final element is the duration, or time axis, for exploring pathways.

### Interacting Helices, as Compositions of 2 Sinusoidal Waves, Converge into an Overall Concentric Helix in the Cylinder Confinement

**Fig. 2:**
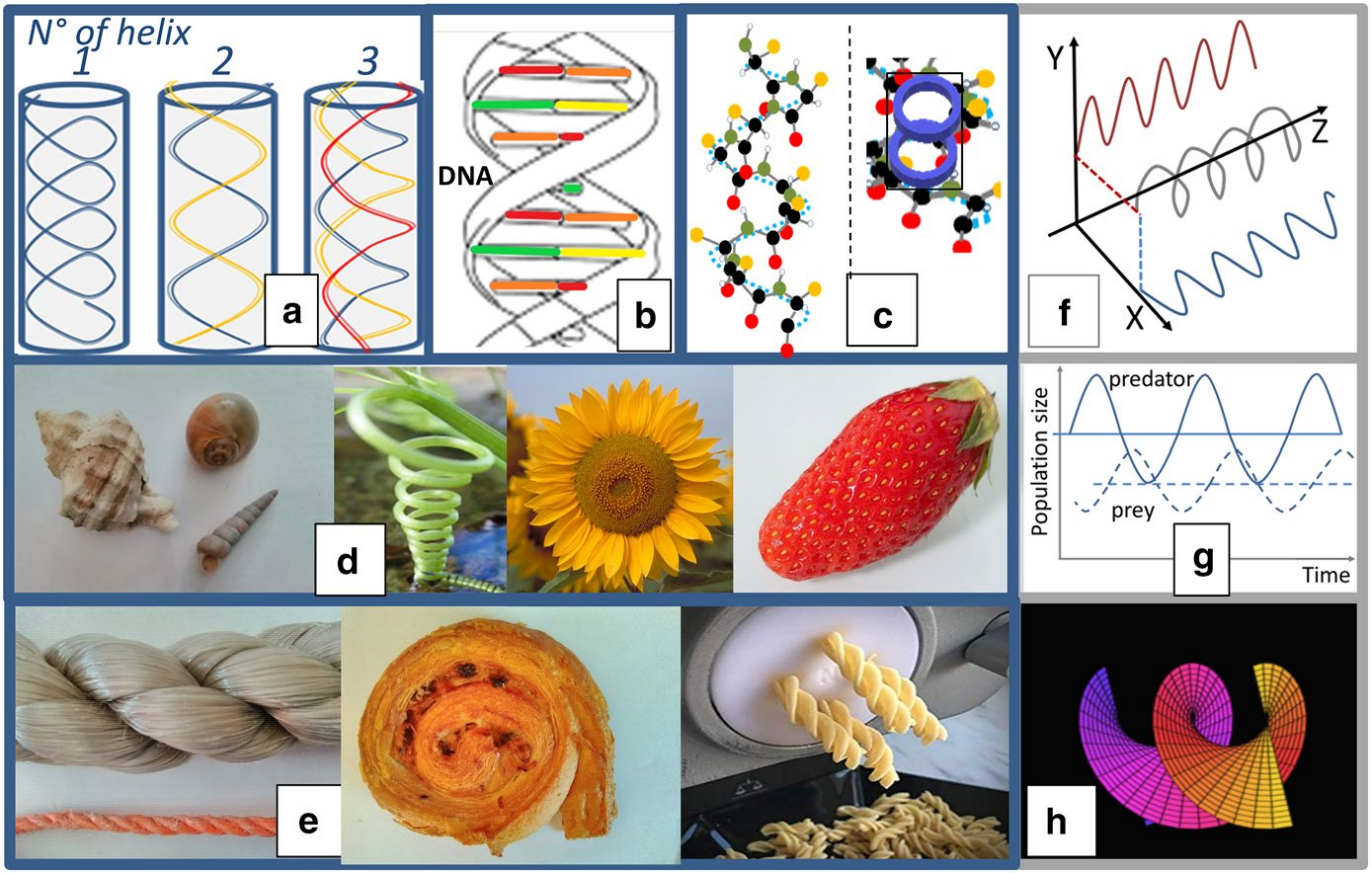
Helical structures; **a** A single, double, and triple concentric helix in the cylinder confinement (modified from Serna et al., 2019); **b** DNA **c** Alpha-helix with top and side views; **d** spiral structures in nature; **e** spiral structures in food and non-food products; **f** two propagating sinusoidal curves form a helix; **g** predator–prey populations in time; **h** helicoid structure. *Source:* Fig. 2a—Helices in a tube, own design, based on work of Serna et al., 2019; Fig. 2d—Spiral growing plant, wikipedia commons; https://commons.wikimedia.org/wiki/File:DirkvdM_natural_spiral.jpg; Fig. 2f—Helix – 2 sinusoidal curves: own design, based on picture of wikipedia commons image: https://www.radartutorial.eu/06.antennas/pic/zirkulanim.gif; Fig. 2g—Own design, inspired by https://en.wikipedia.org/wiki/Lotka%E2%80%93Volterra_equations#/media/File:Lotka_Volterra_dynamics.svg; Fig. 2h—Helocoid: slightly modified, based on https://en.wikipedia.org/wiki/File:Helicoid.svg. From Wikimedia Commons, the free media repository

Does the cylinder confinement enable systems to evolve in a sinusoidal-like manner? Yes, one can learn from fluid dynamics that under specific thermodynamic conditions and considering interacting (nano-)particles with short-range attraction and long-range repulsion, cylindrical confinement promotes the formation of helical structures (Jung & Ha, 2019; Serna et al., 2019). Their morphology depends on pore size and boundary conditions (Fig. 2a). If a short-range attraction is absent, only repulsive interactions govern the system; this behavior is not observed. Helices have mathematically well-described characteristics and are known as essential natural compound structures like those of DNA (Fig. 2b) and of proteins (see for example the α-helix from a top and side view; Fig. 2c). One also recognizes helix structures in living species (Forterre & Dumais, 2011) like plants, shells, sunflowers, and strawberries (spiral structures, following Fibonacci sequences, Fig. 2d). In food and bio-based product processing, stable helices are also rather easily created (see Fig. 2e for ropes, cakes, and pasta).

Helices are among the elementary shapes that are observed in the filamentary and molecular structures of nature. Even though, they are quite complex from mechanical properties points of view and minimum energy configurations (Chouaieb et al., 2006). The conformation of proteins is directed towards the lowest free energy. Although the overall conformation of each protein is unique, two regular folding patterns are often found in parts of them, one is the alpha-helix structure (Alberts et al., 2002). The formation of helical structures is also far from trivial, as shown for example in the food domain for gelatin via covalent functionalization (Zaupa et al., 2011).

For our discourse, it is important to note that, mathematically speaking, *helices*^2^* are three-dimensional structures formed by 2 sinusoidal waves* propagating in time (z-axis) in two perpendicular planes (one sinus in the x–z plane and one in the y–z plane). This is presented in Fig. 2f, and implicitly in Fig. 1 via the dotted curves. If one supposes that the x-axis represents ‘utilization of pieces (either resources, products or services)’, the y-axis the ‘behavior of players’ and the z-axis the ‘time’ (see Fig. 1), then endless propagating helical structures should be formed by needs and behaviors following balancing, sinusoidal-like patterns in between order and chaos. Remarkably, this three-dimensional configuration allows connecting the behavior of stakeholders with the utilization of products in a harmonic way. One could notice similarities between the here presented balancing curves and patterns for predator and prey populations in time within a specific territory, as shown in Fig. 2g. The boundaries are set by socially-accepted norms, ecological boundaries, and economic constraints, hence, the dimensions of the three pillars of sustainability. These social norms, boundaries, and *constraints should always have both lower* – the social foundation guaranteeing the fulfillment of human primary needs – *and upper limits* – the social ceiling avoiding excessive behavior and extreme inequalities – as mentioned before, to present an operational window for actors (or utilized products) in a bioeconomy system to endlessly propagate in a balancing way.

As outlined above, the normative dimensions of SBS are now appearing in the form of a conceptual cylinder framework; in other words, the normative dimensions of sustainable bioeconomy seem to reveal ‘cylindrical symmetry’ in mathematical terms. This implies that ethical leadership, responsible research and innovation programs, decisions of policymakers, or ethical behavior of other players should lead to clearly defined social, environmental, and economic foundations and ceilings. They also should provide acceptable options to continuously balance in the melting zone, here phrased as the safe and fair operating space (Fig. 1).

One could ask if the cylinder framework is also relevant in the case of multiple, interacting, local bioeconomy systems. Figure 2a shows the *co-evolution of multiple, intertwining, helices finally converging into an overall concentric helix*, under well-defined thermodynamic conditions. The helicoid representation of the overall concentric bioeconomy system is in particular interesting to underline, since these are forms of minimal surfaces when bounded by a closed space, like a spiral staircase; this also holds for catenoid structures and planes (Colding & Minicozzi, 2006; partly based on Meusnier J.B. work in 1776; Fig. 2h). Even if such a scheme is a simplification of reality, it serves to explain how multiple local SBS could interact, show co-evolutionary pathways and form the overall, global, concentric SBS.

### Helical Structures in Cylinders, Complex Adaptive Systems, and Open Thermodynamic Systems

**Fig. 3:**
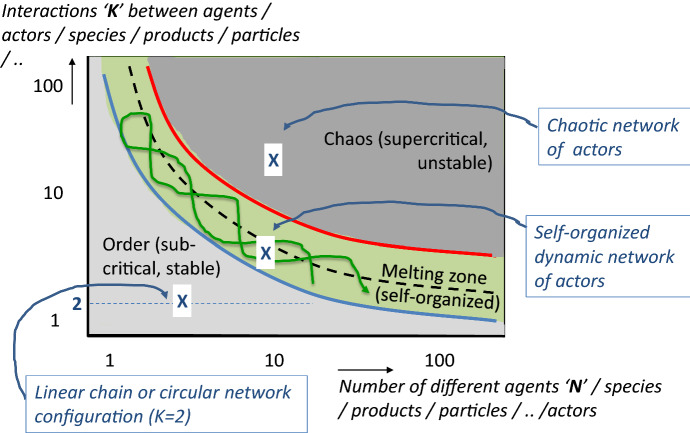
The ‘CAS’ scheme shows four different configurations of actors in bioeconomy systems, either in the order or chaos or melting/sustainability zone; in the latter complex adaptive systems evolve

In our conceptual cylinder framework, the adaptation capacity and the resilience of SBS is lacking. This is of crucial importance for our debate on normative dimensions and ethical considerations concerning the behavior of players. So far, the *complex adaptive system* (CAS; Gell-Mann, 1994; Holland, 1998; Kauffman, 1995) theories are rarely mentioned and not yet systematically explored for integral SBS (de Vries, 2017), except for specific domains like e.g. climate-smart food villages (Jagustović et al., 2019). CAS is based on the thermodynamic plot in which the diversity of N agents (or actors or stakeholders) versus their interactions K is sketched in 2-dimensions, see Fig. 3 (Kaufmann, 1995; de Vries et al., 2018). Herein, three zones are shown: an order, melting, and chaos zone. The melting zone is in between the order and chaos zones, i.e. the same green zone as in the ‘cylinder configuration’ in Fig. 1. It is the zone of self-organization, auto-catalytic behavior, adaptation, and resilience (Martin & Sunley, 2007), emergence, and co-evolution. Co-evolution describes the coupling of fitness landscapes such that adaptive moves by one player deform the fitness landscapes of its immediate partners; this is similar to the interacting individual helices of Fig. 2a resulting in the helicoid in Fig. 2h. In the melting zone, CAS maintains quasi-equilibrium states^3^ (Nicolis and Prigogine, 1989; Kauffman, 1996). Properties include non-linear behavior, scalability, and butterfly effects (Carbonara et al., 2010). The latter implies that minor changes at sensitive intervention points may either maintain the self-organized behavior of actors or push the overall system over the edge of order and chaos (Farmer et al., 2019). This notion is highly relevant today in the discourses about COVID-19. The edges and the bandwidth of the melting zone are determined by external constraints and rules, e.g. acceptable climate conditions, a certain level of (bio-/cultural-) diversity, social limits for the actors, and resource usage. Each constraint – environmental, social, economic – has both a lower and an upper limit in between which a system balances as mentioned before.

*The NK model* of Kauffman (1995), described as the number of interactions ‘K’ between diverse players ‘N’, teaches us the following. If the NK model is applied for either a *purely* linear or circular food chain configuration (number of interactions ‘K’ = 2, i.e. each stakeholder ‘N’ deals with only one supplier and one customer; see Fig. 3; bottom left), the configurations are rigid. If applied for multiple cascading business configurations (very high number of interactions ‘K’ between various stakeholders ‘N’; Fig. 3; top right), the configurations are chaotic. However, if utilized for interactive networks of stakeholders with an intermediate number of interactions ‘K’ between stakeholders ‘N’ (Fig. 3; center), then the configurations become self-organized, showing sinusoidal patterns. The linear and circular concepts may become sustainable in only exceptional cases e.g. if the number of stakeholders becomes very high (N very large; see Fig. 3). The same holds for cascading business pathways with appropriate sets of relatively low N, K-values. This is an important observation for debates about the circular economy, the linear chains for food and other bio-based products, and the cascading concepts for the valorization of resources. Quite often it is stated that strategies are in development to make them sustainable, however, this isn’t always possible as explained here.

Finally, a model for the utilization of products, either as raw agro resources or as processed bio-based products, or services in the melting zone is required (see Fig. 1) which allows revealing sinusoidal-like curves. This model needs to take into account the huge variety of production schemes for renewable resources, the multitude of conversion pathways to assembled products, and the heterogeneity of recyclable products. Second, external inputs are to be considered in all those steps, such as (solar) energy, water, minerals, and gaseous molecules. Third, the potentially unavoidable losses are to be addressed such as heat and the emission of greenhouse gases (GHG). Fourth, the temporal storage or buffering capacity of soils and marine environments for nutrients are to be included. Finally, the planetary lower and upper boundaries (see the introduction and Rockström et al., 2009) require attention like the carrying capacity of the planet (Szekacs, 2017), the limitations for greenhouse gases, and the maintenance of nature and biodiversity. Such a model is thus quite complex. However, a simplified first-order ‘input – moves – output – moves – input – …’ model suffices in the context of our conceptual cylinder framework. We call this here an *Open Thermodynamic and Circular Model (OTCM)*, because of the solar energy input as the driving force, and as outputs the recycled nutrients, unavoidable emissions, and temporal storage of (bio-)matter.

If appropriately and ethically (Blok, 2018) steered, this simplified model allows sinusoidal patterns in the utilization of resources and products to evolve between limits. This implies that mass consumption, avoidable waste streams, over-exploitation of the planetary capacities, GHG emissions above limits, hunger, and poverty, etc. are thus unacceptable options leading to either chaos or order. Responsible research and innovation pathways are foreseen to counteract these options (von Schomberg, 2013). This suggests e.g. that science becomes part of the societal contract (De Saille, 2015) and companies voluntarily carry societal responsibility as expressed in their Corporate Social Responsibility (CSR) reports (Luhmann & Theuvsen, 2016).

### Case Studies

To *exemplify* the conceptual framework, we have selected and analyzed cases in Europe in which we have been involved. These cases cover at least seven system elements: (i) a relatively well-defined playing field, (ii) a group of interacting actors, (iii) specified resources and products, (iv) conversion schemes for resources up to ‘consumed’ bio-based products, (v) rules and constraints like regulations, subsidies and external changing conditions, and (vi) a notion of win-lose. The element ‘duration’ is implicitly present via the starting date of the case. This may be extended for all cases to 2050, which is the time horizon for the Green Deal.

In our experience, the number of case studies covering all elements is rather limited in the bioeconomy literature; most cases are focused on one or only a few system elements. The here selected case studies have not been set up with system or game theories in mind before. They are only selected as they contain sufficient information to exemplify the conceptual framework and to get a notion of its applicability. Future case studies preferably address a larger scope, higher level of complexity, strategic behavior of players, and a territorial setting that encompasses a certain geographical scale, a wide range of actors, pieces, moves, rules, and (intermediate) win/lose outcomes. However, for a first test of the usability of the *cylinder-helix-CAS-OTCM-conceptual framework–in short, conceptual cylinder framework*–the case studies already have their purpose (see Table 1).

**Table 1:** The observations for seven elements of a system or a game (horizontal) presented for nine different cases (vertical)

Element Bioeconomy Case & motivation	Playing field & Time/indicated starting date	Interacting actors / players	Resources and / or products / pieces	Conversion steps / moves	Rules / constraints	Win / lose
Local **manure** and agro-waste initiative *Entrepreneurial initiative to initially transform waste in bioenergy, partly supported by subsidies* (NOAW, 2020)	A community in Germany, *since 2000*	Farmer and owner of a biogas plant, local farmers, technology supplier, own logistics, Town Hall, citizens	By-products and agro-waste: pig, cattle and horse manure, vegetables, and energy crops	Recycling, bioenergy conversion, bio-fertilizer manufacturing	Odor limits, public subsidies, the time limitation for feed-in tariffs	Valorization of over 10,000 tons of organic waste/year New products and markets for local producers; some new jobs created. Public–private cooperation model
New cooperative ‘from cost to value for **wine** by-products’ *Joint cooperative initiative to transform waste into bio-based products, partly supported by subsidies* (NOAW, 2020)	A region in France *since 1970*	Union of wine cooperatives specialized in food and non-food oriented products based on wine by-products Wine producers, Manufacturers, R&D institute, Cooperatives	By-products from wineries and wine processing, nutraceuticals (grape seed extracts), sugars, natural colorings, animal nutrition, alcohol & spirits	Recycling, manufacturing of feed, food, and cosmetics, distilling and extracting constituents, optimizing logistics	Changing regulations for the treatment of wine waste and by-products	Valorization of over hundreds of thousands of tons of grape marc, of Hls of lees and wine must per year; all collected by-products are valorized, no ultimate waste (except process wastewater) Social: over 100 jobs Economic: Almost no cost for wine-makers
Local cluster for by-product valorization from **cereals** *Actors strongly attached to a region, utilizing resources sustainably (foundation initiative)* (NOAW, 2020)	A region in France, *since 2015*	Association as a facilitator to create cross-sector cooperation between cereal farmers/huskers and the eco-construction sector	Processed cereal by-products such as husks into insulation products (in bulk: for floors and ceilings) and into panels for decoration purposes	Enabling cooperation, information, and training of people in using by-products from hulled grain with added value	Fundraising necessary (the chain is not yet mature enough for receiving public support)	Environmental: building with eco-materials, by-products valorization Social: create new jobs in a specialized construction sector A shared passion for by-product valorization
**Food & Energy** Circular economy concept *A network of actors motivated to create an agro-industrial ecology concept for sustainably exploiting resources* (NOAW, 2020)	A community in Germany, *since 2015*	County (biogas plant), Consulting and service provider, University (macroalgae project), Partner specialized in humus, local farmers	Diverse by-products, products such as biogas, electricity, heat, nutrients, humus, dried herbs, barbecue briquettes based on coconut shells	Recycling and bioenergy conversion, developing, and testing modular “add-ons” for biogas plants	The strong impact of “tank-vs.-plate-discussion” resulted in substituting energy crops with horse manure. Question of leadership	Valorization of energy crops, horse manure, and residues from food production; re-use of nutrients as fertilizer. The waste heat from the biogas production is used for drying and cooling herbs Circular economy concept and joint efforts
**Agropark-**industrial-logistic concept *Valorizing an unexploited territory as agro-park* (NOAW, 2020)	A region in The Netherlands *Since 2000*	Agro-business park as new territorial industrial ecology cluster, vegetable producers (greenhouses), traders and data-centers, logistic suppliers	Combined heat, electricity, and water recirculation systems	Closing energy and material circles: Data-centers using electricity from greenhouses and produce heat for greenhouses	Favorable political context: strong promotion of circular economy in The Netherlands	Closed water and energy circles and use waste as much as possible. Some of the greenhouse companies are also involved in projects to create tomato packaging from tomato leaves Membership model showing adherence
Low energy high-quality processing and cooking experience (**NovelQ**); EU project context providing ideas (de Vries & Matser, 2011)	A foodservice context in Europe, *Since 2010*	Technology supplier, cooks, consumers, R&D institute (also involved in Restaurant of the Future alliance)	A large variety of food products (fish, meat, vegetables, etc.) in initially fresh or frozen conditions	Processing, handling, and cooking activities in home situations, canteens, and restaurants (defrosting, heating, consumption)	Low energy input systems, practical implementation, healthy and convenient diets, provision of awards, subventions	Factor 2–10 reduced cooking time (down to minutes) and energy reduction over 50%, while products remain fresh after cooking. Entrepreneurial spirit and inter-SME cooperation
**CEET** consortium for stand-alone, low-energy-autonomous storage and transport container; *An initiative to link ecology, economy, and technology; partly subsidized. The project is later renamed* (CEET, 2013)	Global food chains and local production and storage complexes; potentially also in developing countries for in-field storage, *since 2000*	Climate conditioning firm, solar energy supplier, logistic provider, auctions, energy expert center, R&D institute	Perishable products including flowers, flower bulbs, fruit, vegetables, potatoes, convenience ready-to-eat-meals, etc	Packaging and storage in stand-alone autonomous containers, handling and distributing fresh products at a low energy input	Substantial reduction of waste via closed chains and stand-alone systems, subventions, legal responsibility. No insecticides	Energy reduced over 50%, a breakthrough innovation in intelligent dynamic storage No insecticides due to appropriate climate control (and green chemicals applied exceptionally) New business alliance
**Flexiprocess** A sustainable field to fork concept for mixed protein products; *partly supported by subsidies* (Monnet et al. 2019)	Agro-ecological production fields for wheat and legumes with manufacturing & consumption sites; *since 2015*	Farmers (wheat and legumes), technology suppliers, pasta makers, bakers, consumers, health experts, R&D institute	Cereals, legumes, pasta, pizza, bread, pastry	Producing via novel rotation / agroecological principles, flexible fractionating and manufacturing concepts, consuming and digesting novel food products	Initiative zero pesticides and reduced fertilizers, consumer appreciation, healthy nutrition guidelines	Reduction of fertilizers, healthy soils, new healthy and sustainable diets (divers plant-based protein profiles), biodiversity in resources
Alternative **plantproteins** *A European-driven actor program to motivate enterprise for bio-based action: partly supported by subsidies* (BBI 2020)	Agricultural production fields and manufacturing environment with high by-product streams, *since 2015*	Farmers, manufacturers, technology upgrading company, new ingredients firm, R&D institute	By-products that are rich in proteins like leaves, side-streams from cereals, vegetables, etc	Extracting, separating, and stabilizing proteins for new in-field usage and new product applications	Zero loss in fields & industry; efficient usage of planetary resources; healthy plant-based protein diets	10% unused by-products potentially valorized; local employment; healthy & sustainable alternatives for (partly) animal-protein foods

The bold values are used for the NAME of each case, that is later on systematically used in the main text

## The Conceptual Cylinder Framework Exemplified by Case Studies

### Nine Cases Characterized for Seven Elements of Systems (or Games)

Nine case studies originating either from European, Mediterranean, or national projects have been selected in which the authors have been involved either as an initiator, a (sub-)project coordinator, an advisor, or a researcher. To further test the framework, other described cases may be explored (BBI, 2020; Stadler & Chauvet, 2018). For each case, the presented first observations for the seven system elements have been extracted from project proposals, reports, deliverables, website communications, scientific publications, field visits, and/or discussion meetings with project partners. It should be noted that the observations are here not serving for in-depth analysis and presentation of each case. The latter has been the purpose of (scientific) publications within a European Horizon 2020 project for the first 5 cases, of the final publishable report of a European Six Framework Program project for the 6th case, of the website information of the 7th and 9th cases, and the scientific publication for the 8th case. Our analysis is restricted to (i) verifying if specific data can be presented for each system element, (ii) which information is available about players and their interactions (Fig. 3), (iii) how products are utilized, and (iv) how cases evolve in time (exploiting Fig. 1).

The majority of cases are oriented towards new valorization pathways for agricultural by-products and waste via new business models, innovative technologies, and facilitating public measures. Consequently, the cases address the challenge of the sustainable usage of pieces (resources, products, and services; see Fig. 1) by a group of actors interacting in a concerted way (see Fig. 3). Also, the groups of actors contribute to a revalorization of territorial bioeconomy assets, often in a circular economy context, even though the latter can be questioned from a sustainability point of view (see the remarks about the NK model for bioeconomy above). Others are responding to public measures (‘rules’) by reducing external inputs in agriculture, processing, or in storage and transport (‘win’). Some are reacting to unhealthy diets (a ‘loose’). The observations are summarized in Table 1.

### Interactions Between Players Following the CAS Scheme in Fig. 3

For each case, interactions between actors have been analyzed regarding their positioning in the order-sustainability-chaos scheme of Fig. 3. Overall, the rules and constraints are recognized as clear and transparent; also, the potential scenarios are well-defined as either ‘win’ (‘1′) or ‘lose’ (‘2 & 3′) outcomes of evolving systems responding to specific rules. The ‘Agropark’,’Wine’, ‘CEET’, and ‘Manure’ cases are rather mature, self-organized, dynamic, and autonomously evolving in the melting zone. Agropark and CEET are propagating in the melting or sustainability zone already for quite some years showing a dynamic but stable business behavior. ‘Wine and Manure’ are still not fully stabilized for various reasons like lack of agro resources, larger sourcing areas, and strong competition with manufacturers of conventional products. Hence, Wine and Manure can be positioned at the edge of the order and melting zones.

The NovelQ and PlantProtein examples are near-mature with a strong business potential, competitive advantages due to unique and partly protected technologies and wide application areas. However, they continue to work on a sound and stable technology and business model.

The Food&Energy and Cereals cases are promising examples for the future, however, actually in immature phases. The geographical playing fields are well-defined. The business and technology models are currently in development. There is a need for new actors and interactions, to cross the frontier of the rigid and melting zones.

Flexiprocess is fully dependent on the support of external parties and yet immature as a business. However, as a more food-chain technology-oriented concept it already has an added value as research has shown. Their positioning is still in the rigid zone.

### Balanced Utilization of Products via the OTCM Coupled to the Conceptual Cylinder Framework

If one considers the simplified OTCM for products, the cases are in general showing a balanced utilization of resources and products within the green melting zone of the conceptual cylinder framework. This is in particular due to circular flows of biomass, efficient input of energy and water (key examples are Agropark and Food&Energy), prioritizing valorization of by-products above loss and accumulation of waste (Manure, Wine, Cereals, Plantprotein), and lowering energy input in conversion and handling steps (NovelQ, CEET). This is further supported by the change towards resources that are demanding fewer inputs, the provision of more sustainable diets (Flexiprocess), the exploration of territorial production capacities without striving for the usage of external inputs while respecting seasonal fluctuations (Wine, Cereals). It should be noted that all cases intend to optimize the production and utilization of resources and products in a well-balanced way.

### Combined Results for the Conceptual Cylinder Framework and Capacity to Respond to Changes

The results show that modifications in one element, like technological (moves) or organizational (player) innovations, or the introduction of new rules are always leading to modifications in the other elements. This is needed to reach a favorable outcome. For example, for the new valorization steps of by-products, also new business models are emerging (e.g. Food&Energy). For a new territorial organizational innovation (e.g. Agropark and Flexiprocess), new connections for flows of biomass are established between actors. Another rule-incentive (e.g. changing subventions in the case of Manure) generates a new business model and production of alternative added-value products. This means that elements are interconnected, however, not yet per se sustainably. This should be tackled in forthcoming research and innovation projects focusing on a more in-depth understanding of all interconnected elements, appropriate indicator sets, and variable constraints settings like in Game Theory.

It should be noted that the selected cases do not allow analyzing inter-dependencies between playing fields, hence looking to the integration of helices into a helicoid structure for an overall, planetary, bioeconomy system (see discussion for Fig. 2a). This remains to be done in the next studies.

## Discussion

This section deals with (i) the need for a new conceptual framework for Sustainable Bioeconomy Systems (SBS), (ii) a detailed discussion of the cylinder framework, its applicability and understanding of sustainability in a dynamic system, and (iii) the potential of the framework to study interacting (local) systems.

(i) While the first and second bioeconomy strategies and agendas of the European Commission have been product- and biotechnology oriented (Bugge et al. 2016), the third is more an ecological and circular bioeconomy strategy. These strategies do not guarantee that the global and local bioeconomy systems are sustainable from an environmental, social, and economic point of view. Neither is the understanding of the positive or conflicting intentions from public and private stakeholders to strive for sustainable solutions based on a well-founded theory. This makes all debates about the normative dimensions of the bioeconomy systems so complicated and challenging. Here, we have attempted to provide a unifying conceptual framework as the theoretical basis for these debates. This framework underlines the challenge and importance of a coherent and integrative approach based on concomitantly addressing the seven system or game theory elements in a cylinder configuration, the interactions (K) between diverse agents (N) according to CAS-theory, and balanced production and utilization of products and resources following the OTCM. As compared to previous 2-D configurations like the radar and doughnut, the here introduced cylinder configuration allows visualizing and understanding pathways of SBS avoiding entering in chaos or rigidity zones. The cylinder configuration also permits to link the seven key elements of systems (or games), namely playing fields, players, pieces, moves, rules, outcomes, and duration.
(ii) A deeper insight into the 3 zones (order, chaos, and melting or sustainability or safe and fair operating space) is obtained via complex adaptive system theories. These theories are partly founded on thermodynamic laws, in particular on the graph describing the diversity of agents versus their interactions leading either to order, chaos, or self-organized behavior. Intuitively it is known that if many diverse actors are all interacting with each other, the outcome is chaotic (like for all people speaking at meetings at the same moment). Also, if there are no interactions or nearly no actors, the outcome is highly static or rigid (like for nobody saying a word at a party). Somewhere in between the discussions make sense and are dynamic. As explored by Kauffman (1995), these interactions can be mimicked by Boolean networks with N different agents and K number of interactions. In a network of ‘N’ stakeholders with a reasonable amount of interactions ‘K’, bioeconomy systems are becoming self-organized, dynamic with overall sinusoidal behavior (Fig. 3; core). Endless growth of economies due to increasing N-K values will undoubtedly lead to chaos (Fig. 3; top right). Consequently, these economies will be ethically hard to defend if not additional considerations like pressure valves (Zwier et al., 2015) are introduced to equilibrate systems. Passive approaches and inactivity will lead to rigidity and highly static food systems (see Fig. 3; bottom left).
  The unifying conceptual framework is not complete if the notion of the planet as an open thermodynamic system is not taken into account. This implies that there is a driving force (solar energy), several conversion steps, and a re-balancing output (stored biomass or emitted particles or infrared radiation). In a very simplified way, this is described as OTCM. The consequence is that an ideal circular concept doesn’t make sense; dynamic systems follow helical pathways.
  As shown above, the cylinder framework seems to be useful for analyzing diverse bioeconomy cases. Most importantly, it reveals that elements are interconnected. If ‘interacting actors’ and ‘product utilization’ both show sinusoidal patterns and are interfering, in well-defined playing fields, with transparent rules, and appropriate moves (conversion steps), then the overall outcomes are ‘wins’. The patterns reveal helical pathways in the zone between order and chaos, i.e. following scenario 3 in Fig. 1. If one of the behavior patterns shows steady growth (or decline) patterns, then the overall outcome is ‘lose’, the system ends up in chaos (scenario 1) or rigidity (scenario 2). Systems are sustainable if they follow scenario 3, and unsustainable for scenarios 1 and 2.
  The systems following scenario 3 have a certain capacity to respond to external changes like increasing levels of agro- and food waste in chains, loss of nutritional quality, new incentives, and excessive energy inputs as shown by our cases. For example, changing subsidies in the Manure or banning of insecticides in the CEET cases have led to new organizational and technological innovations and sound business cases. However, it finally depends on the resilience and adaptation capacity of bioeconomy systems, their capacity to find new equilibria whether or not this also holds for climate change, drought, viruses like COVID-19, biodiversity loss, financial crisis, new laws, etc.
  The unifying conceptual framework may help in better understanding how sustainability pathways could be reached, which are leverage points and (remaining) constraints. As an illustration and based on the insights obtained in our cases and framework, some very first and preliminary suggestions are provided here:
  The connection of bioeconomy systems with agroecology (Caquet et al., 2020) may be the only way to make the agroecology transition sustainable. If the sustainable evolution of new food and bio-based products, sustainable behavior of all interacting actors, and adopted rules in the full system are not taken into account, the agroecology transition may enter either the order or chaos zones (Fig. 1).
  Valorizing entire plant resources and waste streams via cascading schemes may be beneficial from a resources efficiency point of view, however, may unintentionally lead to chaos (see Fig. 3); the number of interactions between (new) actors in the cascading systems should be taking into account and being ‘manageable’; also policy measures are to be considered to guarantee self-organized dynamical solutions and sustainable outcomes.
  The choice of playing field dimensions – like for urban metabolisms (Bezama et al., 2019) – should very critically be re-considered because sustainable outcomes may never be reached in these playing fields; hence, interactions with other playing fields (nearby territories like rural-coastal areas) and/or external players and resources should then be taken into account. The ‘ensemble’ of playing fields, via fair and compensating exchange mechanisms for all system elements, should be sustainable. The considerations about uncompensated unsustainability measures in one playing field, and leaving the burdens to other playing fields, are ethically and scientifically unjustifiable.
  The circular bioeconomy in itself is highly rigid (Fig. 3), except if the number of actors in the circle is extremely high. The business model typology for local circular economy initiatives (Donner et al., 2020) shows in majority network-models revealing 6 different cooperation types between robust but flexible actors striving for integrated usage of resources locally. To avoid rigidity, insights in resilience and adaptation capacity are crucial, especially when subsidies are altered for resource usage or new European competition rules are introduced for interacting SMEs (Gerbrandy & de Vries, 2011).
(iii) Our 9 selected cases do not yet show the interactions and trade-offs between different local bioeconomy systems. This is very important in the case of e.g. sustainable urban bioeconomy systems (see above) or for food sovereignty as currently debated. For example, if one country or region strives for autonomous supplies to feed its population, what are the consequences for other countries? Which mutual support options should be put in place to keep systems overall viable and ethically justified (like emergency aids, migration, and additional support externally)? And should each local system be sustainable as our framework strongly suggests? What does this imply for the normative dimensions of both the overall system and local systems? One can draw a parallel with an orchestra of individual musicians: the overall outcome could only be harmonic (like the helicoid structure in Fig. 2a) in case all individual contributions are harmonic.

## Conclusions

The here presented unifying conceptual cylinder framework allows analyzing and discussing diverse SBS. This is in a very preliminary way done via the 9 cases, and provides the following conclusions:

- Without a coherent conceptual frame, including the time axis, coherent sets of both lower and upper limits for social, environmental, and economic indicators, and a linkage between the 7 key system or game theory elements, one cannot distinguish if systems are sustainable or not. *Continuous growth (or decline)* strategies – and related discourses – are automatically *leading to chaos or rigidity* and are ethically and scientifically not justified. Feedbacks and interventions in the 7 elements are necessary to keep balancing outcomes in between order and chaos. This implies that *interventions always include counter-balancing actions* to keep the system within boundaries (Fig. 1), expressed by Latour as ‘Down to Earth’ (2017).
- Disrupting or innovating one element (e.g. technology, business, regulation, or social) impacts the other six elements. If this destabilizes systems and results in unsustainable outcomes, then *integral measures for all elements are required* (like today discussed for climate change, biodiversity loss, COVID-19, etc.). If not, individual and mono-disciplinary, single domain or sector-oriented interventions make sense for local optimizations in the system.
- Here, the conceptual cylinder framework reveals that (i) player behavior and usage of biomass sustainably are interconnected and should jointly follow helical pathways to be sustainable (see Fig. 1), (ii) the helicoid, as the sum of individual helices representing co-evolving local systems, is the lowest energy (thus preferable) state for SBS at our planet (like DNA or protein triple helices in nature) (cf. Figure 2), (iii) the top view of the cylinder reveals a circle which currently serves as the basis for circular economy thinking, however without the time dimension and thus without the possibility to analyze sustainable evolutions, (iv) helical structures are highly stable but dynamic structures in nature, hence they are perfectly well suitable for being resilient and adaptive to external shocks.

**Fig. 4:**
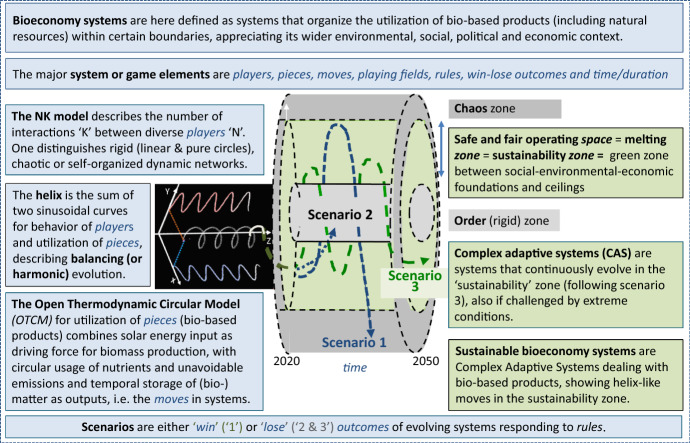
*Synthesis* of the major building blocks of the Conceptual Cylinder Framework for Sustainable Bioeconomy systems. *Source:* Modified image of https://www.radartutorial.eu/06.antennas/pic/zirkulanim.gif is included

Overall, the *unifying conceptual cylinder framework (theory) now permits us to define Sustainable Bioeconomy Systems as Complex Adaptive Systems dealing with food and other bio-based products, showing helix-like moves in the sustainability zone.* This is due to the coherence between Complex Adaptive System, NK models, open thermodynamic models, helical moves in cylinders between order and chaos, and the exploitation of system or game theory elements; a synthesis is presented in Fig. 4. This integrally includes playing fields, dynamically interacting (existing and new) actors, (novel) bio-resources and products, all eco-friendly conversion steps, critical conditions and rules, duration and timings, and win-lose scenarios which are crucial for finding sustainable solutions and steering (policy) measures.

The notion of territories as evolving playing fields requires further reflection because their characteristics are modified by the dynamics of the (replicating) agents themselves. *The traditional way of considering fixed country or regional borders as political choices may be inadequate for considering territories from a sustainability point of view.* Those should encompass e.g. geographical, atmospheric, political, or sectorial dimensions to provide freedom to sustainably operate within limits. Hence, an intelligent re-design of playing fields, adapted case studies, and the analysis of external conditions – arriving from other ‘playing fields’ – are imperative for concluding about all possible interactions in the systems and ethically justified sustainable outcomes (wins).

Finally, one could suppose that the *normative dimensions of the bioeconomy have cylinder symmetry*. Such a cylinder symmetry enables bioeconomy systems – constituted of different interacting players utilizing bio-based products in well-defined playing fields – to endlessly evolve in a balanced or sustainable manner in the melting zone between order and chaos (scenario 1 in Fig. 1), hence, respecting boundaries. In other words, the cylinder framework also provides a direction to ethical, philosophical, and esthetical debates in the bioeconomy.

## Data Availability

All references are given.

## Footnotes

1 *EC,* 2011; ‘*Over the twentieth century, the world increased its fossil fuel use by a factor of 12, whilst extracting 34 times more material resources. Today in the EU, each person consumes 16 tons of materials annually, of which 6 tons are wasted, with half going to landfill. Whilst demand for food, feed and fiber may increase by 70% by 2050, 60% of the world’s major ecosystems that help produce these resources have already been degraded or are used unsustainably.’.*

2 It should be noted that our representation of a helix is not referring to the helix systems described by Carayannis et al., (2019). The latter are linking knowledge, innovation and environment, either as ‘university-industry-government relations’ (triple helix), an additional “media-based and culture-based public” (quadruple helix) and a final extension to the inclusion of the “environment” (quintuple helix).

3 Not a permanent equilibrium, which would not allow survival of species.
